# Understanding context in the implementation of emergency obstetric and neonatal care in health facilities in Osun State, Nigeria- a mixed-methods study

**DOI:** 10.1186/s12884-022-05278-7

**Published:** 2022-12-13

**Authors:** Abiola Olubusola Komolafe, Oyeyemi Olajumoke Oyelade, Sunday Adepoju Adedini, Omolola Oladunni Irinoye

**Affiliations:** 1 Department of Nursing Science, Obafemi Awolowo University, Ile-Ife, Nigeria; 2 Demography and Social Statistics Department, Federal University Oye-Ekiti, Oye-Ekiti, Nigeria; 3 Demography and Population Studies Programme, Schools of Public Health and Social Sciences, University of the Witwatersrand, Johannesburg, South Africa

**Keywords:** Context, Implementation, Emergency obstetric and newborn care, Referral facilities

## Abstract

**Background:**

Successful implementation of Emergency Obstetric and Neonatal Care (EmONC) is likely to improve pregnancy outcomes and is essential for quality maternity care. Context in implementation is described as factors that enabled or disabled implementation of interventions. While the context of implementation is important for the effectiveness of evidence-based interventions, the context of EmONC implementation has not been widely studied in Nigeria.

**Methods:**

The research design was cross-sectional descriptive. A mixed-methods approach was used to assess and explore the context of implementing EmONC in referral centres in Osun state. A purposive sampling technique was used to select the three tertiary health facilities in Osun State and six secondary health facilities from the six administrative zones in the State. A total of 186 healthcare providers in these referral centres participated in the quantitative part of the study, and eighteen in-depth interviews were conducted for its qualitative aspect. An adapted questionnaire from Context Assessment Index and an interview guide were used to collect data. Quantitative data were analysed using descriptive and inferential statistics at 0.05 significance level, while qualitative data were analysed using the thematic approach.

**Results:**

The percentage mean score of context strength in EmONC implementation was 63% ± 10.46 in secondary and 68% ± 10.47 in tertiary health facilities. There was a significant difference in the leadership (F (1, 184) = 8.35, *p* < 0.01), evaluation (F (1, 184) = 5.35, *p* = 0.02) and overall context (F (1, 184) = 6.46, *p* = 0.01) of EmONC implementation in secondary and tertiary health facilities. Emerging themes in EmONC context were: Resources for EmONC implementation; Demand for EmONC; Efficiency of funding; Institutional leadership; and Performance evaluation.

**Conclusions:**

The context of EmONC implementation in the referral health facilities was generally weak. The secondary health facilities’ weaknesses were worse compared to the tertiary health facilities. The five key contextual factors could inform strategies for improving EmONC implementation in health facilities to ensure improved access to care that will reduce deaths from obstetric complications in Nigeria.

**Supplementary Information:**

The online version contains supplementary material available at 10.1186/s12884-022-05278-7.

## Background

Context of implementation of an intervention or programme in healthcare can be described as the totality of factors that enable or disable an organisation from implementing. Previous studies affirm that context is a significant determinant of implementation of complex interventions [1–5]. Context could be defined ‘as a set of unique factors that come into play in an implementation effort [6]. The context of implementation is thus key to the effectiveness of evidence-based interventions in real-life practice.

Global attention to maternal and newborn health indicators, including mortality, morbidity and women’s experiences of care, prompts the need to take into account the implementation of evidence-based interventions [7, 8]. Although EmONC is an evidence-based intervention to avert deaths from obstetric complications, its effectiveness may vary in different contexts. Its implementation in health facilities in Nigeria has not yielded optimal results, as many maternal and neonatal deaths still happen. For over five years (2011–2015), Nigeria had a maternal mortality ratio of 814/100,000 live births (range 596-1,180) [9]. A multisite study involving referral hospitals reported higher hospital-based MMR (2,085/100,000 live births; range 877–4,210) in 2014 [10]. The neonatal mortality rate was equally high and increased from 34/1000 births in 2015 to 38/1000 births in 2018 [11]. Maternal and neonatal deaths in Nigeria were higher than in many other low-income countries. Many more women, however, experience childbirth-related morbidities and undignified care in Nigeria [12–14]. High maternal mortality and morbidity suggest EmONC may not have been successfully implemented in health facilities, which may be associated with the context of its implementation [10].

Nigeria’s health system is driven by the public and private sectors. More than two-thirds of health facilities are public and funded by the Local (primary level), State (secondary level) and Federal governments (tertiary level) [15]. The two complementary types of EmONC facilities are basic EmONC and comprehensive EmONC healthcare facilities [16]. Inequities, however, in geographical locations and allocation of resources in favour of secondary and tertiary health facilities have paralysed many primary healthcare facilities. With this background, many pregnant women bypass primary health facilities to seek basic care in higher-level health facilities, which made these facilities provide basic and comprehensive EmONC [15, 17].

Taking context into consideration as a factor in implementation has been emphasised in the literature. From a broad theoretical explanation, the context consists of inner and outer settings [6] and proximal and distal factors [18]. Outer settings consist of political and economic factors, while inner settings are the structure and culture [6, 19]. Proximal factors in the context of care include staffing patterns, types of healthcare providers available, and availability of resources to less proximal factors such as availability of guidelines based on current evidence [6, 18]. Other essential but distal factors include policies regulating the scope of practice, delineating what each healthcare provider can do and the type of medications administered at various health system levels [18]. Evidence from a systematic review of contextual factors influencing implementation suggests that availability of resources such as funds, time, staff and training enhances implementation [20].

As Nigeria failed to achieve the Millennium Development Goals (MDGs 4 & 5) to reduce child mortality and improve maternal health, this study assessed culture, leadership and evaluation of the overall context of EmONC implementation in secondary and tertiary health facilities and tested for contextual differences between the two levels [21].

## Methods

The Integrated Promoting Action on Research Implementation in Health Services (I-PARIHS) framework was adopted as the theoretical framework to investigate context of EmONC implementation. It explains factors that enable or disable implementation of interventions and determine implementation success [22–24]. I-PARIHS specifies three main elements of context: culture, leadership and evaluation [22, 23]. The context assessment index is an objective tool for assessing context based on culture, leadership and evaluation, similar to the elements in I-PARIHS [25]. Culture could be described as the manner activities are carried out in health facilities, and leadership denotes the organisational structure, control and guidance. Evaluation involves using evidence to inform decisions on the effectiveness of evidence in practice.

### Study design and setting

The research design was cross-sectional descriptive. A concurrent mixed methods approach was used to assess and explore the context of care in implementing EmONC in the secondary and tertiary health facilities in Osun State, South-west Nigeria. The State has three senatorial districts, divided into six administrative zones and thirty-one Local Government Areas (LGAs). There are nine secondary and three tertiary health facilities. The State has the highest percentage of facility-based births (89.1%) and births guided by skilled providers (94.2%) in Nigeria [26].

### Study population and sampling

Purposive sampling technique was used to select nine LGAs from the six administrative zones in the State. Selection was based on the availability of tertiary and secondary (State hospital) health facilities in the LGAs. The target population included doctors, nurse-midwives and facility managers working in the maternity units. A total of 186/228 (81.6%) healthcare providers in the maternity units consented to participate in the quantitative part of the study.

### Data collection

#### Quantitative

A pre-tested, structured, self-administered questionnaire, adapted from the Context Assessment Index (CAI), which measured the context on a 4-point Likert scale, was used to collect data [25, 27]. A pilot study was conducted among a similar population as the study population to determine the validity and reliability of the questionnaire before its use in this study. Some of the items in CAI which needed to be better understood by the healthcare providers, lowering the reliability of the questionnaire, were either reframed or removed, and some items were included based on the qualitative data. The reliability coefficient of each context element (culture *r* = 0.90, leadership *r* = 0.76, evaluation *r* = 0.74) and the overall context questionnaire (*r* = 0.93) was computed from the pilot study, while validity was established through face and content validity.

#### Qualitative

The qualitative study was designed to explain the context of EmONC implementation from the perspectives of healthcare providers and facility managers who are implementers of EmONC. Participants were selected from those who participated in the quantitative study. One healthcare provider and one facility manager from each of the nine facilities were interviewed based on their consent to participate. Inclusion criteria for healthcare providers were having at least three years of clinical experience in the maternity unit and providing direct care to pregnant women with obstetric complications. The exclusion criterion was being a trainee-specialist doctor. Trainee-specialist doctors were excluded from the interview because they were still in training, and the power structure in the administration of the facilities may interfere with their responses. The facility managers were the heads of maternity units in the facilities. Equal numbers of males and females in the qualitative study were possible because all facility managers were males and all healthcare providers coincidentally were nurse-midwives and females.

A semi-structured interview guide was used to conduct in-depth interviews. These took place in private places in maternity units to ensure confidentiality. The decision to interview one healthcare provider and one facility manager from each secondary and tertiary facility enabled saturation. Collection of qualitative data from different sources (nurse-midwives who were healthcare providers and facility managers who were also medical practitioners) ensured triangulation and maximum insight in the context of EmONC implementation. The interviews, which lasted 45–60 min, were tape-recorded. Interview transcripts were presented to the interviewees to check if the transcripts were true accounts of the interviews to ensure credibility. This, in a way, contributed to the validity of the findings. Also, the study being part of a Ph.D. project enjoyed peer debriefing with significant contributions for improvement and, being a mixed method study, benefitted from different methodological approaches to investigate the context of EmONC implementation.

### Data analysis

#### Quantitative data analysis

Context is measured with 28 questions on a 4-point Likert scale of 1 (strongly disagree), 2 (disagree), 3 (agree) and 4 (strongly agree). Culture has fourteen questions items, leadership six, while evaluation has eight. Data showed normal distribution with the Shapiro-Wilk test for normal data (0.77) and the Kurtosis test for normality (0.66). Thus parametric tests were performed. Mean scores < 3 were regarded as ‘weak context’ while ≥ 3 as ‘strong context’. Percentage mean scores for culture, leadership, evaluation and overall context were computed by multiplying the mean score by 25 so that each context element was expressed in percentage, representing the strength of the context of EmONC implementation. Percentage mean scores < 75% were adjudged ‘weak context’, while scores ≥ 75% were adjudged ‘strong context’. Descriptive data analysis was done using frequency, percentage, mean and standard deviation, while inferential statistics used ANOVA.

#### Qualitative data analysis

Audio-taped interviews were transcribed verbatim. Qualitative data analysis was done independently by the first and second authors. Data analysis was done alongside data collection, and saturation was reached when there was no other perspective to understand context of EmONC. Data were organised into nodes in NVivo, and themes were identified and clustered to achieve a level of abstraction needed to interpret and explain the context of EmONC implementation in the referral health facilities.

Strengthening the Report of Observational Studies in Epidemiology (STROBE) checklist for cross-sectional studies was used [28].

## Results

Mean age of the respondents in the health facilities was 38 years ± 8.47. The majority were female, 91.8% in secondary and 79.6% in tertiary facilities. Also, majority were nurses, 81.6% in secondary and 62.8% in tertiary facilities (Table 1).

**Table 1:** Respondents’ socio-demographic and occupational characteristics by type of health facility (*n* = 186)

Variables	Secondary (*n* = 49) Freq (%)	Tertiary (*n* = 137) Freq (%)
**Age: Mean = 38.68 ± 8.47**	**41.78 ± 10.39**	**37.04 ± 7.31**
20–29	7(14.3)	22(16.1)
30–39	14(28.6)	66(48.2)
40–49	15(30.6)	43(31.4)
50 and above	13(26.5)	6(4.4)
**Sex**		
Male	4(8.2)	28(20.4)
Female	45(91.8)	109(79.6)
**Educational level**		
Diploma	20(40.8)	30(21.9)
First degree	29(59.2)	107 (78.1)
**Job category**		
Medical practitioner	9(18.4)	51(37.2)
Nursing staff	40(81.6)	84(62.8)
**Years spent in the maternity unit Mean 2.5 ± 1.33**	**3.0 ± 1.46**	**2.1 ± 1.03**
Less than one year	9(18.4)	39(28.5)
1–3 years	15(30.6)	58(42.3)
4–6 years	7(14.3)	27(19.7)
7–9 Years	7(14.3)	7(5.1)
10 years and above	11(22.4)	6(4.4)

Respondents had mean scores < 3.00 in all items in the culture context except in “high regard for patient’s privacy and dignity” in both secondary and tertiary facilities (Table 2). Mean scores of items in culture context were higher in tertiary than in secondary facilities except in three items: (1) Clinical leaders help to remove barriers to changing practice in maternal & child care; (2) High regard for patient’s privacy and dignity; (3) Development of staff expertise is viewed as a priority by clinical leaders. Mean scores of item ‘Provision of sufficient support for EmONC by government and local authorities were 2.51 ± 0.79 in secondary and 2.50 ± 0.82 in tertiary facilities (Table 2).

**Table 2:** Respondents’ culture context in EmONC in secondary and tertiary health facilities

Culture context	Secondary	Tertiary
**Culture**	**2.68 ± 0.48**	**2.79 ± 0.43**
Conducive environment to develop and share ideas	2.90 ± 0.79	2.96 ± 0.65
Patients feedback on care is encouraged	2.84 ± 0.74	2.93 ± 0.69
Performance review process is in place which enables reflection on EmONC practice, goal setting	2.61 ± 0.88	2.80 ± 0.69
High regard for patients’ privacy and dignity	3.27 ± 0.63	3.12 ± 0.66
Appropriate information on EmONC (in large prints, tapes, etc.) is accessible to patients	2.47 ± 0.84	2.55 ± 0.85
Development of healthcare providers’ expertise is viewed as a priority by clinical leaders	2.86 ± 0.70	2.80 ± 0.67
Staff use reflexive process (e.g. clinical supervision) to evaluate and develop practice	2.73 ± 0.86	2.93 ± 0.69
In this organization, all necessary resources are available to deliver EmONC	2.22 ± 0.74	2.37 ± 0.81
Facility management provides professionals with training to deliver EmONC	2.23 ± 0.80	2.71 ± 0.74
Goals and outcome for implementing EmONC are communicated with healthcare providers	2.76 ± 0.75	2.85 ± 0.72
Clinical leaders help to remove barriers to changing practice in maternal & child care	2.94 ± 0.86	2.84 ± 0.73
Provision of sufficient support for EmONC by government and local authorities	2.51 ± 0.79	2.50 ± 0.82
National Health Insurance Scheme (NHIS) covers basic emergency obstetric and neonatal care	2.59 ± 0.91	2.88 ± 0.75
National Health Insurance Scheme (NHIS) covers comprehensive emergency obstetric and neonatal care	2.39 ± 0.93	2.80 ± 0.83

Mean scores in all items of the leadership context were higher in tertiary than in secondary facilities (Table 3). Mean scores < 3.00 were observed in all items in the leadership context in both secondary and tertiary facilities (Table 3).

**Table 3:** Respondents’ leadership context in EmONC in secondary and tertiary health facilities

Leadership context	Secondary	Tertiary
**Leadership**	**2.32 ± 0.49**	**2.58 ± 0.54**
The management structure is democratic and inclusive	2.55 ± 0.70	2.69 ± 0.80
Management is willing to listen to problems with delivering EmONC following the guidelines.	2.06 ± 0.55	2.45 ± 0.78
Management is helpful with delivering EmONC following guidelines.	2.20 ± 0.67	2.45 ± 0.81
Counting on management support when things get tough with EmONC	2.20 ± 0.70	2.42 ± 0.76
I have a clear plan of how I will deliver EmONC following the guidelines.	2.02 ± 0.80	2.52 ± 0.82
All staff, both medical and nursing have an equal opportunity to participate in decisions regarding EmONC	2.90 ± 0.92	2.93 ± 0.80

Mean scores in all items of the evaluation context were higher in tertiary than secondary facilities (Table 4). Mean scores < 3.00 were observed in all items in the evaluation context in both secondary and tertiary facilities (Table 4).

**Table 4:** Respondents’ evaluation context in EmONC in secondary and tertiary health facilities

Evaluation context	Secondary	Tertiary
**Evaluation**	**2.57 ± 0.46**	**2.73 ± 0.42**
Healthcare providers are empowered to develop practice	2.82 ± 0.78	2.82 ± 0.75
Structured education programs are available for all HCPs	2.65 ± 0.83	2.74 ± 0.72
Clinical leaders act as role models of good practice in EmONC	2.94 ± 0.82	2.90 ± 0.64
Audit and feedback are used to develop EmONC practice	2.73 ± 0.94	2.83 ± 0.65
Health facility management has regard for staff autonomy	2.61 ± 0.75	2.69 ± 0.69
My role in delivering EmONC following the guidelines are clearly defined for me	2.00 ± 0.68	2.52 ± 0.84
Professionals with whom I deliver EmONC deliver EmONC following guidelines.	1.88 ± 0.60	2.50 ± 0.82
Healthcare providers have strong work relationships that support implementing EmONC	2.90 ± 0.85	2.86 ± 0.69

**Fig. 1:**
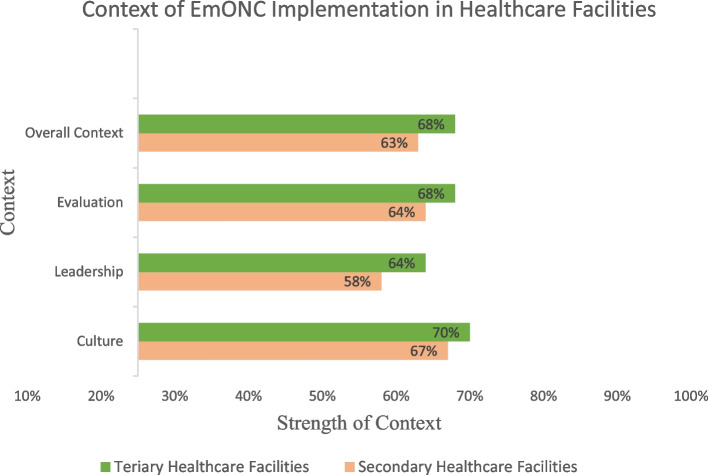
Context of EmONC Implementation in Secondary and Tertiary Healthcare Facilities

Figure 1 shows context percentage mean scores in the secondary and tertiary facilities. The percentage mean scores were < 75% in culture, leadership, evaluation and overall context in both secondary and tertiary facilities.

Statistically significant differences were observed in EmONC implementation in secondary and tertiary facilities for leadership (F 1, 184 = 8.35, *p* < 0.01), evaluation (F 1, 184 = 5.35, *p* = 0.02) and overall context (F 1, 184 = 6.46, *p* = 0.01) in contrast with culture (F 1, 184 = 2.40, *p* = 0.12) (Table 5).

**Table 5:** Difference in EmONC implementation context in health facilities

Domain of Context		Type of Facility	*n*	Mean (SD)	SS	MS	F	*p*-value
Culture Context	Between groups	Secondary	49	2.68 ± 0.48	0.47	0.47	2.40	0.12
	Within groups	Tertiary	137	2.79 ± 0.43	35.91	0.20		
Leadership Context	Between groups	Secondary	49	2.32 ± 0.49	2.20	2.20	8.35	< 0.001
	Within groups	Tertiary	137	2.58 ± 0.54	50.63	0.28		
Evaluation Context	Between groups	Secondary	49	2.57 ± 0.46	0.99	0.99	5.35	0.02
	Within groups	Tertiary	137	2.73 ± 0.42	33.99	0.18		
Overall Context	Between groups	Secondary	49	2.52 ± 0.42	1.13	1.13	6.46	0.01
	Within groups	Tertiary	137	2.70 ± 0.42	32.27	0.18		

### Qualitative data

Table 6 shows respondents’ socio-demographic and occupational characteristics in the qualitative study. All nursing staff in tertiary facilities had a first degree compared to 3 out 6 nursing staff in secondary facilities. Also, all medical practitioners in tertiary facilities had a fellowship. They were specialist doctors in obstetrics and gynaecology, while 5 out of 6 medical practitioners in secondary facilities had a first degree and were medical officers.

**Table 6:** Socio-demographic and Occupational Characteristics of Respondents for IDI (*n* = 18)

Variable	Secondary Healthcare Facilities (*n* = 12) Freq (%)	Tertiary Healthcare Facilities (*n* = 6) Freq (%)
**Sex**		
Male	6 (50.0)	3 (50.0)
Female	6 (50.0)	3 (50.0)
**Educational Level**		
Registered Nurse-midwife	3 (25.0)	0 (0.0)
First degree	8 (66.7)	3 (50.0)
Fellowship in Obstetrics & Gynaecology	1 (8.3)	3 (50.0)
**Job Designation**		
Nursing Staff	6 (50.0)	3 (50.0)
Medical Officers	5 (41.7)	0 (0.0)
Obstetrics & Gynaecology Specialist	1 (8.3)	3 (50.0)
**Role designation**		
Facility manager (FM)	6 (50.0)	3 (50.0)
Healthcare provider (HCP)	6 (50.0)	3 (50.0)

Five major themes emerged from the different perspectives of context. Corresponding categories for each theme are stated in Table 7.

**Table 7:** Emerging Contextual Factors influencing the Implementation of EmONC

Context	Themes	Categories
Culture	Resources for EmONC	• Human resources • Material resources • Technical knowledge resources
	Demand for EmONC	• Belief system • Affordability of EmONC cost
Leadership	Efficiency of Funding	• Politics and policy making (free health versus out-of-pocket payment for healthcare • Government commitment to funding health • Dysfunctional healthcare system
	Institutional Leadership	• Receptive context • Role models for EmONC • Managerial attitude • Locus of decision
Evaluation	Performance Evaluation	• Review and educational meetings • Feedback on cases of obstetric complications • Perceived success in EmONC implementation

### Theme 1: resources for EmONC

Human, material and technical knowledge resources are important dimensions to understanding the context of EmONC implementation. Human resources refer to the number of qualified healthcare providers implementing EmONC, while material resources are drugs, equipment and consumables for implementation of EmONC. Technical knowledge resources address the quality aspect of human resources and refer to all activities geared towards enhancing the competencies of healthcare providers in implementing EmONC.

Views indicated that resources for EmONC have not been sufficient in health facilities. Participants in secondary facilities indicated shortages of healthcare providers, materials needed to provide EmONC and capacity building of healthcare providers to implement EmONC were not given priority. One participant in a secondary facility said:

*‘We don’t have the equipment here……. we don’t have a blood bank. They have to go to a private facility to buy blood and most of the resources needed to handle such complications are not available’* *Female, Nurse, Secondary facility.*

However, the most perceived lack among participants from tertiary healthcare facilities was the lack of materials. One participant said,

*‘The problem we have is not personnel as such, although we have shortage of personnel but the major challenge is delivering our service within a short time, that is the time taken from the arrival of the patient requiring our service and the delivery of appropriate care and this is because we don’t have in place what materials we should have’* *Male, Medical doctor, Facility manager, Tertiary facility.*

Emphasis on the need for more human and material resources for EmONC is a major impediment to implementation. Lack of human resources is more acute in secondary facilities with two nurse-midwives and one or two medical officers (one also being a facility manager) in the morning shift. For afternoon and night shifts, only one nurse-midwife was available per shift with a medical officer on call. At least two nurses were on each shift (morning, afternoon and night) in tertiary facilities. Usually, many doctors worked in the morning and were on call later in the day (two doctors on housemanship, two resident doctors (trainee specialist doctors) and one consultant obstetrician). In essence, staffing patterns are worse in secondary facilities with regard to numbers and quality.

From the perspectives of technical knowledge resources, variations were observed in access to EmONC training and guidelines for healthcare providers in secondary and tertiary facilities. Healthcare providers in secondary facilities lacked access to training and were not using guidelines for implementing EmONC. One nurse-midwife reflected on this:

*‘Emergency obstetric and neonatal care has not been implemented as intended by protocol because there is no training to improve skills and knowledge of health care providers’* *Female, Nurse, Secondary facility.*

However, reports about training and inconsistent use of guidelines for implementing EmONC existed in tertiary facilities. Participants acknowledged EmONC training, although healthcare providers stipulated there had not been any in recent times:

*‘The institution organizes emergency obstetric and neonatal management but for some months now we have not had any training, any formal training. The last one was 3 years ago’* *Female, Nurse, Tertiary facility.*

Another participant said:

*‘I have had training on PPH. It lasted for 3 days, that was about 2 years ago’* *Female, Nurse, Tertiary facility.*

Inconsistency in the use of guidelines was shown in the reports of some participants from tertiary facilities. A facility manager commented:

*‘We have protocols for obstructed labour, PPH, eclampsia and severe pre-eclampsia but in piecemeal’* *Male, Medical doctor, Facility manager, Tertiary facility.*

Although guidelines are essential to ensure uniformity and quality of care, many participants did not have access to EmONC guidelines. The availability of EmONC guidelines piecemeal may not allow healthcare providers to be aware these existed. Lack of continuous training and unavailability or inconsistent use of EmONC guidelines may negatively impact the competencies to implement EmONC in health facilities.

### Theme 2: demand for EmONC

Demand in this context means women patronising health facilities to receive EmONC. Demand is vital in the implementation of any intervention. Demand for EmONC will involve patients’ beliefs supporting EmONC for obstetric complications. It will also require that women give birth in health facilities and those with obstetric complications (and their significant others) make prompt decisions to access EmONC. Participants generally expressed concern about the poor conditions in which women with obstetric complications reach health facilities. They believed that the women’s cultural beliefs and patronage of traditional birth attendants are responsible for the delay in seeking facility-based care that could afford them the opportunity to receive EmONC in case of obstetric complications. One participant said:

*‘Most women, because of their traditional belief, don’t report to the hospital on time. They report to the wrong places like going to the Traditional Birth Attendants (TBAs)’* *Male, Medical doctor, Facility manager, Secondary facility.*

Another participant said:

‘*Most of the cases of Post-Partum Haemorrhage (PPH) we have, have gone to the Traditional Birth Attendants (TBAs), gone to quacks and that has been the main source of the problem. When it becomes a very big problem, they push them out’* *Female, Nurse, Tertiary facility.*

Some assertions, however, show poor perceptions of care rendered in health facilities. That may hinder women from coming to health facilities and prevent the implementation of EmONC for those in need. A participant said:

*‘We have quacks around this place, and the women think if they get there, they will attend to them promptly and they now bring complications’* *Female, Nurse, Secondary facility.*

While culture and traditions are essential in making decisions to seek facility-based care, affordability of costs may be a major impediment to seeking care in the facilities. Many women with obstetric complications may not be able to afford the costs of EmONC. Also, the purchase of healthcare by many women was out-of-pocket. This was agreed to be happening because of the failure of the health insurance scheme and hospital management organisations in either enrolment or purchasing of healthcare. This view was expressed when a participant said:

*‘The patient has to pay out of pocket. Some of those things (materials needed for EmONC) are not available in the hospital because they have not been supplied since they (management) have to pay the suppliers for previous supply. NHIS system, for now, is not working very well from what we are seeing, HMO has refused to refund the hospital for the money paid by patients treated on NHIS. So the management cannot pay their suppliers and suppliers are not supplying materials’* *Male, Medical doctor, Facility manager, Tertiary facility.*

### Theme 3: efficiency of funding

Efficiency in the funding of EmONC requires government commitment to funding health as reflected in budgetary allocations to health. Government politics and healthcare policies are part of the measures for efficient health funding. In reality, efficiency of funding EmONC demands that adequate numbers of qualified healthcare providers are employed to implement EmONC. It also implies sufficient resources are provided to ensure EmONC implementation as intended. Being able to train healthcare providers in EmONC indicates efficient funding. Healthcare providers’ motivation and commitment to EmONC implementation lie in the payment of salary and inducement allowance to healthcare providers and perceived benefits of EmONC for obstetric complications, which are all a function of government commitment to funding health. Efficient funding of health facilities will usually culminate in an optimal functioning health system.

Some views indicate that EmONC implementation needs to be more efficiently funded in health facilities. The policy of free healthcare by the state government was seen as political propaganda as many patients still have to pay for healthcare. Healthcare providers felt that the government established Public-Private Partnership pharmacies to ‘indirectly collect money from patients’. In their opinion, the government should have allowed patients to pay for healthcare and used the revenue generated to equip the hospital with the necessary resources for quality care for mothers and newborns. One participant said:

*‘They are indirectly collecting money from the patient because they incorporated some private pharmacy into the pharmacy unit, and those are the ones selling drugs and materials. So why not let the people pay and let us give them the care they need. Let there be resuscitation gadget, so I believe that this is the greatest factor affecting the health sector’* *Female, Nurse, Secondary facility.*

Participants’ presentations of the situation of health facilities reflect a dysfunctional health system, as resources to implement EmONC are not available. One of them said:

*‘We need funding for EmONC implementation. It gets tough when there was no funding. There is a collapse of the system, they (patients) come and whatever you need to resuscitate and operate is not there’* *Male, Medical doctor, Facility manager, Tertiary facility*.

Participants in secondary facilities said lack of government commitment was reflected in non-payment of salary, resulting in lack of motivation among healthcare providers. One facility manager said:

*‘Even the job motivation is not there on the part of healthcare providers that are already employed by the government, so there might not be enough commitment towards the job’* .

He further stressed:

*‘not the training, not the government policy, it is the satisfaction one gets after seeing a positive result, that is what is keeping us going’* *Male, Medical doctor, Facility manager, Secondary facility.*

Participants believed they do everything to give the best possible care to women with obstetric complications despite inefficient funding.

### Theme 4: institutional leadership

Institutional leadership is essential in creating a receptive context for implementing EmONC, entailing the presence of role models who drive and supervise EmONC implementation in health facilities. Leaders’ educational background, nature of involvement and attitude are important for the implementation of EmONC. Leaders should establish mechanisms that enable the implementation of EmONC, showing commitment to these matters. Such institutional leadership for EmONC was absent in secondary facilities.

Locus of control is essential in the implementation of interventions. Participants from secondary facilities reported centralisation of decision-making, which hindered a receptive context for EmONC implementation. Facility managers did not contribute to decision making, and decisions such as whom to train, when to train and which type of training to give are made outside the facilities and passed down to the managers in charge. Centralisation of decisions hindered EmONC implementation. One manager said:

*‘We don’t have the capacity to send staff for training because we are being controlled by the central body in the state’* *Male, Medical doctor, Facility manager, Secondary facility.*

In contrast, decentralisation of decisions occurs in tertiary facilities. Professional boundaries appeared to be respected. Even though the obstetrician is the head of the maternity unit, the nursing staff are allowed to express themselves in the implementation of EmONC within the constraints of what is permitted by the principles guiding their practice. One participant:

*‘I attended training on neonatal resuscitation…. for doctors and nurses…The message (to attend a training) came through the Deputy Director of Nursing Services’* *Female, Nurse, Tertiary facility.*

Majority of the managers in secondary facilities were medical officers without specialisation in obstetrics, limiting their capacity to act as role models in implementing EmONC One healthcare provider said:

*‘There is no effective communication between the management and the staff…. nobody is supervising anybody; you just do your work’* *Female, Nurse, Secondary facility*.

Managers in tertiary facilities are specialised in obstetrics, acting as role models in EmONC implementation. One participant said:

*‘My superiors supervise me and we also work hand in hand. Anytime I am about to move away from what is right, they will easily call my attention back to doing it right’* *Female Nurse, Tertiary facility*.

### Theme 5: performance evaluation

Performance evaluation includes reflective processes and feedback mechanisms to learn and improve EmONC implementation in health facilities. Some views indicate that performance evaluation needs to be improved in secondary facilities. One participant said:

*‘There are no records for audit and feedback, so how will they audit care? We need to improve on our documentation’* *Female, Nurse, Secondary facility.*

Participants in tertiary facilities had performance review meetings and educational activities in place. They believed such meetings enabled performance evaluation and improved EmONC implementation.

*‘There is morbidity and mortality committee meeting where the heads of the team meet weekly to discuss care rendered to patients. Also, maternal death review is a weekly thing’* *Female, Nurse, Tertiary facility.*

Participants in secondary facilities did not receive feedback on referrals of women with obstetric complications to tertiary facilities. Such feedback could have provided opportunities to engage in reflective processes to learn and improve EmONC implementation.

*‘We had one case of obstetric complication that we think we might not be able to manage. We refer based on that, unfortunately there was no feedback with regard to the woman. We did not know what eventually happened’* *Male, Medical doctor, Facility manager, Secondary facility.*

Investigation of EmONC implementation showed participants have varied perceptions of success. Many participants in secondary and tertiary facilities perceived success in EmONC implementation because they were able to save the lives of many women and newborns. Dissenting voices, however, existed to these claims. One participant said:

*‘No, (EmONC is not successfully implemented) because when we are talking about the implementation like the way teaching hospital (tertiary facility) runs, they have everything on the ground, we too as secondary facilities are supposed to have everything on the ground (like) staff to be enough, equipment on the ground, there should be incentives, regular payment of staff. If all these are on the ground, they will help people to work’* *Female, Nurse, Secondary facility.*

Another participant said:

*‘In my own opinion I wouldn’t say they (EmONC signal functions) have been implemented properly because we cannot tackle the issue of handling third delay which is delay in receiving appropriate care or initiating correct care when the patient gets to the hospital…Until somebody comes to us and we initiate the correct treatment within the appropriate time as soon as possible. It (EmONC) has to be initiated correctly and promptly’* *Male, Medical doctor, Facility manager, Tertiary facility.*

While lack of resources was the major reason for perceived failure in EmONC implementation in secondary facilities, long waiting times and delays in receiving EmONC were reasons for perceived failure in tertiary facilities.

## Discussion

The weak culture of insufficient resources impedes EmONC implementation. Insufficient resources have been identified as contextual barriers to the implementation of interventions and provision of quality care [29, 30]. Knight et al. also reported drug procurement, logistic problems and lack of equipment as the commonest context-related barriers to receiving timely and appropriate care in health facilities [31]. There is lack of EmONC training and guidelines in many health facilities [32]. Referral of women with obstetric complications from secondary to tertiary facilities and even private hospitals could be frustrating for healthcare providers because of lack of resources. Many may quit working in secondary facilities, worsening staff shortages or increasing staff turnover. High staff turnover hinders EmONC implementation because of the frequent need to train new staff, its implication on time and resources and initial loss of progress due to discontinuity [33].

Insufficient resources in health facilities are linked to the weak context of institutional leadership and inefficient funding of healthcare [34, 35]. Leadership is a mediator of other contextual factors influencing implementation success [20]. Leadership in tertiary facilities seems to be better with the presence of obstetricians who act as role models and provide consultation on EmONC, which was absent in secondary facilities.

Facility-based maternity care was low (37%) in Nigeria, also because EmONC information was not made accessible during antenatal care [36]. Women may not receive appropriate information during pregnancy, which may be why they fail to recognise pregnancy-related danger signs and present late in health facilities for maternity care [37–39].

Many women prefer to access care from traditional birth attendants because they have easy access to them and receive care without delay [40]. Long waiting times and bureaucracy witnessed in the Nigerian health system are absent with traditional birth attendants. More so, there is non-confidential and undignified care during childbirth in Nigeria, preventing women from utilising facilities [41].

Inefficient functioning and poor patronage of primary health centres that are supposed to provide basic EmONC to women with obstetric complications also contribute to delays in access to secondary and tertiary facilities. From another perspective, high patronage of traditional birth attendants in communities, even where primary health centres are available, is a big challenge to risk reduction of obstetric complications. Inequities in the location of secondary and tertiary facilities also make women seek care from traditional birth attendants. Again, there is inequity in healthcare affordability and funding in Nigeria. The principle of universal health coverage specifies that all people must have access to the health care they need without financial hardship [42]. Lack of financial protection contributes to denial and delay seeking EmONC by women with obstetric complications. While a country’s health financing strategy determines the extent to which it will achieve universal coverage, Nigeria’s budgetary allocation to health has been grossly deficient. It has never exceeded 50% of the minimally required budgetary allocation [15, 43]. The State government’s political propaganda of free maternal and child health will only result in poor healthcare funding, weak drug supply and inadequate infrastructure [44].

Strategies to finance healthcare in Nigeria include health insurance to pool risks so that individuals can access the care they need without undue financial burden. Although public health facilities are accredited national health insurance centres, health maintenance organisations (HMOs) in Nigeria have yet to sufficiently pool the risks and purchase quality healthcare for the people [45]. Out-of-pocket health expenditure is 70%, well above the 40% regarded as catastrophic health expenditure [43, 46]. National health insurance enrolment is less than 10%, and less than 3% of women of reproductive age are covered [47, 48]. Arguably, the low number of covered women of reproductive age implies that universal health coverage would not be achieved in Nigeria.

There was a weak context of evaluation processes to support EmONC implementation. A learning organisation utilises reflective processes to learn implementation of interventions. Although tertiary facilities have evaluation mechanisms such as maternal and newborn death reviews and educational meetings, this was absent in many secondary facilities. There needed to be feedback from patients, and even tertiary healthcare facilities failed to provide feedback to the secondary healthcare facilities on the referral of women with obstetric complications. Also, audit and feedback processes, which could serve as learning opportunities for healthcare providers, were deficient in health facilities. Providing feedback, however, on implementation to staff increases the rate of successful implementation [49, 50]. Also, lack of an educational program to develop evidence-based practices is a major factor that hinders implementation [51, 52]. Resources and process of implementation are key to the successful implementation of interventions [20, 53–55]. A holistic measure of success in EmONC implementation would consider the resources for EmONC, implementation and maternal and perinatal outcomes such as women’s satisfaction, mortality and morbidities. Many maternal deaths, however, still happen in referral facilities, but more in secondary than in tertiary facilities [8].

## Conclusion

Successful EmONC implementation is vital towards achieving universal health coverage and improved maternal and newborn health. The context of EmONC implementation in secondary and tertiary facilities in our study setting was weak. Although weak, the context of EmONC implementation in tertiary facilities was better than in secondary facilities, which may be responsible for better pregnancy outcomes in tertiary facilities. Findings around the discourse of context of EmONC implementation in both referral facilities showed inadequacies. Therefore, needs exist to intensify efforts to improve these contexts for better maternal and neonatal outcomes in Nigeria.

## Supplementary Information

**Additional file 1**. Adapted Context Assessment Questionnaire.

## Data Availability

The datasets generated and/or analysed during this study are not publicly available because the study was part of a larger study, but are available from the corresponding author on reasonable request.

## Abbreviations

EmONC: Emergency Obstetric and Neonatal Care
MMR: Maternal Mortality Ratio
MDGs: Millennium Development Goals
LGAs: Local Government Areas
CAI: Context Assessment Index
NHIS: National Health Insurance Scheme
HMO: Health Maintenance Organization
PPH: Postpartum Haemorrhage
