# Supplementary material for: Understanding context in the implementation of emergency obstetric and neonatal care in health facilities in Osun State, Nigeria- a mixed-methods study
Source: BMC Pregnancy Childbirth. 2022 Dec 13;22:934. doi: 10.1186/s12884-022-05278-7 (PMC9746005; doi:10.1186/s12884-022-05278-7)
Supplement: Supplementary file 1 — Additional file 1. Adapted Context Assessment Questionnaire. [file 12884_2022_5278_MOESM1_ESM.docx]

**Adapted Context Assessment Questionnaire**

**Section A: please, fill in the gap as appropriate**

1. Date of birth: __________________________________
2. Sex: Male ( ) Female ( )
3. Level of Education: Diploma () First degree () Masters () PhD () Others, specify __________
4. How would you describe your professional job category? Facility manager ( )

Medical practitioner ( ) Nursing staff ( ) CHEW( )

1. Ward/Unit: ____________________________________
2. Years of experience: ____________________________
3. How many years have you worked in this ward or unit? Less than one year ( ) 1-3 years ( ) 4-6 years ( ) 7-9 years( ) 10years and above ( )

**Section B: The following statements relate to context of implementation of emergency obstetric and newborn care. Please indicate the extent you agree or disagree with the statements**

| S/N | Item | *STRONGLY AGREE* | *AGREE* | *DISAGREE* | *STRONGLY DISAGREE* |
| --- | --- | --- | --- | --- | --- |
|  | Clinical nurse leaders create an environment conducive to the development and sharing of ideas |  |  |  |  |
|  | Patients are encouraged to participate in feedback on care |  |  |  |  |
|  | Facility management provides professionals with a training to deliver EmONC |  |  |  |  |
|  | Goals and outcome for implementing EmONC are communicated with staff |  |  |  |  |
|  | Clinical leaders help to remove barriers to changing practice in maternal and child care |  |  |  |  |
|  | Government and local authorities provide sufficient support for EmONC |  |  |  |  |
|  | NHIS covers basic emergency obstetric and neonatal care |  |  |  |  |
|  | NHIS covers comprehensive emergency obstetric and neonatal care |  |  |  |  |
|  | Performance review process is in place which enables reflection on EmONC practice, goal setting & is regularly reviewed |  |  |  |  |
|  | There is high regard for patients privacy and dignity |  |  |  |  |
|  | Appropriate information on EmONC in large prints are accessible to patients e.g. in  print, tapes, etc. |  |  |  |  |
|  | Development of staff expertise is viewed as a priority by  clinical leaders |  |  |  |  |
| S/N | Item | *STRONGLY AGREE* | *AGREE* | *DISAGREE* | *STRONGLY DISAGREE* |
|  | Staff use reflective processes (e.g. action learning, clinical supervision) to evaluate or develop practice |  |  |  |  |
|  | In this organization, all necessary resources are available to deliver EmONC |  |  |  |  |
|  | The management structure is democratic and inclusive |  |  |  |  |
|  | The management of the organization I work in is willing to listen to my problems with delivering EmONC following the guidelines. |  |  |  |  |
|  | The management is helpful with delivering EmONC following the guidelines. |  |  |  |  |
|  | I can count on support from management of organization I work in, when things get tough using guidelines |  |  |  |  |
|  | I have a clear plan of how I will deliver EmONC following the guidelines |  |  |  |  |
|  | All staff, both medical and nursing have an equal opportunity to participate in decisions regarding EmONC |  |  |  |  |
|  | HCPs feel empowered to develop practice |  |  |  |  |
|  | Structured programs of education are available to all HCPs |  |  |  |  |
|  | Clinical leaders act as role models of EmONC following guidelines |  |  |  |  |
|  | Audit & feedback are used to develop practice of EmONC |  |  |  |  |
|  | Health facility management has high regard for staff autonomy |  |  |  |  |
|  | My role in delivering EmONC following the guidelines are clearly defined for me |  |  |  |  |
|  | Professionals with whom I deliver EmONC deliver EmONC following guidelines. |  |  |  |  |
|  | HCPs have strong work relationships that support implementing EmONC |  |  |  |  |

Thank you for sparing some time to respond to the questionnaire.
